# Detection of Small-Sized Insects in Sticky Trapping Images Using Spectral Residual Model and Machine Learning

**DOI:** 10.3389/fpls.2022.915543

**Published:** 2022-06-28

**Authors:** Wenyong Li, Zhankui Yang, Jiawei Lv, Tengfei Zheng, Ming Li, Chuanheng Sun

**Affiliations:** ^1^National Engineering Research Center for Information Technology in Agriculture, Beijing, China; ^2^College of Computer Science and Technology, Beijing University of Technology, Beijing, China; ^3^College of Information Science and Technology, Zhongkai University of Agriculture and Engineering, Guangzhou, China; ^4^College of Information, Shanghai Ocean University, Shanghai, China

**Keywords:** pest detection, sticky trap, small objects detection, image processing, machine learning

## Abstract

One fundamental component of Integrated pest management (IPM) is field monitoring and growers use information gathered from scouting to make an appropriate control tactics. Whitefly (*Bemisia tabaci*) and thrips (*Frankliniella occidentalis*) are two most prominent pests in greenhouses of northern China. Traditionally, growers estimate the population of these pests by counting insects caught on sticky traps, which is not only a challenging task but also an extremely time-consuming one. To alleviate this situation, this study proposed an automated detection approach to meet the need for continuous monitoring of pests in greenhouse conditions. Candidate targets were firstly located using a spectral residual model and then different color features were extracted. Ultimately, Whitefly and thrips were identified using a support vector machine classifier with an accuracy of 93.9 and 89.9%, a true positive rate of 93.1 and 80.1%, and a false positive rate of 9.9 and 12.3%, respectively. Identification performance was further tested *via* comparison between manual and automatic counting with a coefficient of determination, *R*^2^, of 0.9785 and 0.9582. The results show that the proposed method can provide a comparable performance with previous handcrafted feature-based methods, furthermore, it does not require the support of high-performance hardware compare with deep learning-based method. This study demonstrates the potential of developing a vision-based identification system to facilitate rapid gathering of information pertaining to numbers of small-sized pests in greenhouse agriculture and make a reliable estimation of overall population density.

## Introduction

Integrated pest management (IPM) has been widely applied to the agricultural practices in the field to minimize yield loss and reduce the use of chemical insecticides (Boissard et al., 2008; Espinoza et al., 2016; Rustia et al., 2020). This approach utilizes underlying presence of natural enemies, or likelihood of presence in the field (Wen and Guyer, 2012; Yang et al., 2021). Therefore, the accurate detection of pest species is essential for maximizing the successful IPM.

In greenhouses, one of the most common approaches used for pest detection is using sticky traps to capture insects and subsequently count the presence (and number) of target pest species on these traps. Based on the density and severity of pests in the greenhouse, growers apply appropriate control tactics (Ebrahimi et al., 2017). However, traditional manual identification and counting of insects on a trap is a time-consuming and labor-intensive task. Given these underlying challenges associated with the identification and counting of insect pests in the greenhouse, an automatic pest detection approach is vital to the modern agricultural production.

With advancements in imaging technology and computer software, image-based approaches have been developed in recent years for the detection of small-sized pests in greenhouse agriculture, including traditional machine learning and deep learning methods. In the term of traditional machine learning, Solis-Sánchez et al. utilized shape features (e.g., eccentricity and area) and adaptive threshold discriminant method to detect whiteflies (Solis-Sánchez et al., 2010). To improve feature robustness, they extracted invariant features to discriminate and identify different insect species and an improved precision was achieved compared to previous work (Solis-Sánchez et al., 2011). Besides, Xia et al. (2012) introduced a multifractal analysis approach for detecting whiteflies on a sticky trap *in situ* using a mobile robot to collect insects. Furthermore, to improve pest counting efficiency, Xia et al. (2015) proposed an automatic pest identification method suitable for long term monitoring *in situ* with less computational cost by applying YCbCr color space for segmentation and Mahalanobis distance for identification of pest species (Xia et al., 2015). Espinoza et al. proposed an image processing system that involved object segmentation, as well as morphological and color property estimations, to detect whitefly and thrips (Espinoza et al., 2016). However, these color-based object segmentation methods were not robust to various conditions in the field, such as variable illumination and sticky glue degeneration. Rather than directly counting the pests captured on the traps, Sun et al. presented a counting algorithm to treat trapped pests as “noise” in a two-dimensional (2D) image with two-dimensional Fourier transform (2DFT) serving as a specific noise collector (Sun et al., 2017), but it could not separate pests from real environmental noises and thus did not resolve the species identification problem. In contrast to conventional machine learning methods, deep learning methods automatically ascertain the comprehensive features from the training dataset, avoiding complex image processing procedures during object segmentation and labor-intensive feature engineering to meet various outdoor conditions. Rustia et al. developed a cascaded approach that detects and filters out non-insect objects from the detected objects using a convolutional neural network (CNN) detector in the first stage and then further classifies the obtained insect objects into different species using a multi-class CNN classifier (Rustia et al., 2020). Li et al. (2021) proposed a deep learning model on the basis of the Faster R-CNN architecture to optimize the detection accuracy of tiny pests in sticky trap images from agricultural greenhouses.

Although the above-mentioned studies have achieved good performance and solved some special problems, there is still space for improvement in this area of research. For instance, these methods based on traditional machine learning are not flexible due to the object segmentation bases on threshold strategies. In deep learning area, the typical classification models using the CNN structure rely on large datasets to train the models, but actually, it is hard to obtain a large labeled dataset in many cases (Li and Yang, 2020). Furthermore, greenhouse pests such as whitefly (*Bemisia tabaci*) and western flower thrips (*Frankliniella occidentalis*) are small in size, which will cause information loss during the multi-layer convolution in deep learning architecture. Although many object detectors based on deep learning perform well on medium and large objects, they perform poorly on the task of detecting small objects (Tong et al., 2020). This is because small objects lack appearance information needed to distinguish them from background or similar categories. However, comparing to image background, these tiny pests could be regarded as many “novelty” objects in the sticky trapping images. Since the spectral residual model is independent of features, categories, or other forms of prior knowledge of the objects, it has been widely in small object detection (Zhou and Zhang, 2007; Cui et al., 2012; Deng and Duan, 2013). Therefore, we investigate whether it can be also applied to detect very small pests under natural greenhouse conditions.

In this study, we propose a spectral residual model-based method in combination with a support vector machine (SVM) classifier to identify the most important pests in greenhouse of northern China, namely whitefly (*Bemisia tabaci*) and thrips (*Frankliniella occidentalis*). This work provides a major step toward population estimation in greenhouses and providing accurate, rapid and reliable results to aid in decision making processes for pesticide application and pest management approaches.

## Materials and Methods

### Data Collection

**FIGURE 1:**
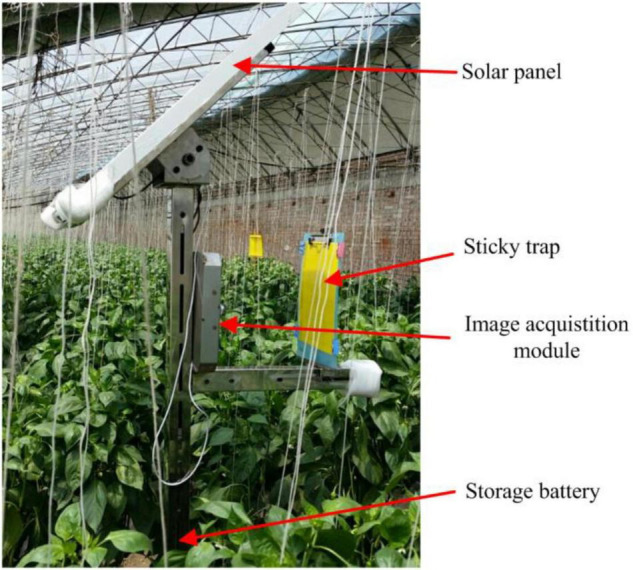
Image acquisition equipment and sticky trap for detection of insect pests in greenhouse conditions (Li et al., 2021).

Red-green-blue (RGB) color images were captured automatically by a pest monitoring device (Figure 1) in a greenhouse located in Fangshan district, Beijing, China (39°38′19.29″N, 116°01′29.98″E). The device consisted of a solar panel, sticky trap, image acquisition module and storage battery. The device was deployed in the center of the greenhouse, and the height of the sticky trap (25 × 30 cm, Pheorbio^®^) was above the crop at 1.5 m from ground level. The sticky trap is a typical attractant trap used widely for collection of pests of interest whereby insects became adhered to the sticky surface. The experiment was carried out on green pepper plants cultivated under greenhouse conditions.

Two species, adult-stage whitefly (*B*. *tabaci*) and thrips (*F. occidentalis*) were selected as the detection target in this study. Solid-color traps were used to avoid “noise” in the digital images caused by grids, as previously reported elsewhere (Xia et al., 2015; Espinoza et al., 2016). Images of the sticky trap (25 × 20 cm) were collected and transmitted to a remote server at 2,560 × 1,920 pixels every 2 h daily (8:00 a.m. to 18:00 p.m.). Generally, the sticky paper is replaced every 6 days to maintain good trapping effectiveness. Therefore, in this study, eighteen original images were selected to extract training samples from six consecutive days, that is, three original images were selected each day in the period (one image in the morning, midday, and afternoon, respectively). Likewise, eighteen original images were selected to create test samples from another six consecutive days. Thereafter, sample images of three classes, two target species and background, were extracted with a square box of 32 × 32 pixels manually from the original images. Ultimately, 500 sample images for each class, totally 1,500 sample images, were randomly selected from the first eighteen original images to construct the training dataset. And all target species (whitefly and thrips) on the second eighteen original images were used as test dataset.

### Detection Method

The proposed detection method consisted of three stages: candidate object location, feature extraction and multi-class recognition. The candidate object location is a pipeline to detect the location of objects (section “Candidate Object Location”), feature extraction devotes to extract feature of the detected objects (section “Feature Extraction”) and these obtained objects were then further classified into whitefly, thrips and background in the stage of multi-class recognition (section “Multi-Class Recognition Model”). These procedures are outlined in the following subsections.

#### Candidate Object Location

**FIGURE 2:**
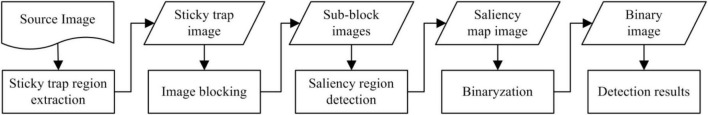
Flow chart of the candidate object location pipeline from source image to detection results.

Before performing feature extraction and pattern recognition, the locations of candidate targets within the image are determined. The locationpipeline in the sticky trapping images involved several subroutines, as shown in Figure 2. First, a color-based segmentation approach is design to extract the sticky paper region from the original image. Then, the sticky trapping image is divided into sub-block images and objects in each sub-block image are locally detected using a saliency region detection model. Subsequently, a threshold is determined and used to obtain the location of the objects.

##### Extraction of Sticky Paper Region

**FIGURE 3:**
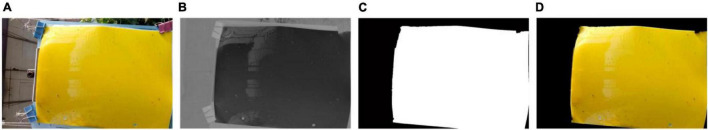
Illustration of the sticky trap region extraction using image processing technology: **(A)** original image, **(B)** Cb component in YCbCr color space, **(C)** binary image, and **(D)** extraction result of the specific region of interest.

The sticky paper region, denoted as the region of interest (RoI) in this study, is extracted from the original image. First, the original image (Figure 3A) is transformed into YCbCr color space from the RGB color space and the RoI could be distinguished from background based on the Cb component of YCbCr color space (Figure 3B). Subsequently, the Cb component is processed into a binary image (Figure 3C) using the Ostu method (Otsu, 1979) and a morphological fill operation. Finally, the RoI image (Figure 3D) is obtained by performing a logical conjunction between the original image (Figure 3A) and the binary image (Figure 3C).

##### Image Blocking

**FIGURE 4:**
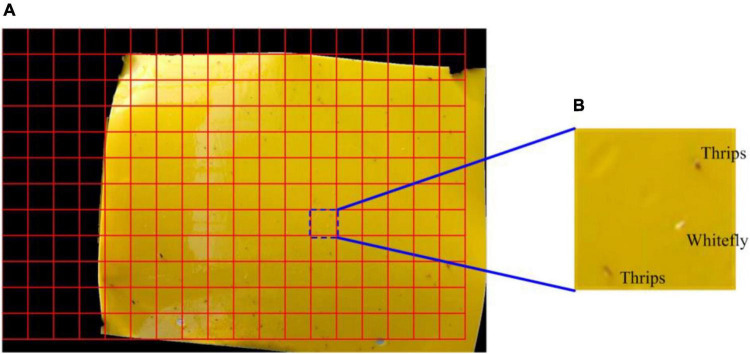
Image blocking diagram. **(A)** This sticky trapping image is divided into a specific region of interest with a specific scale and **(B)** an illustration of an enlarged sub-block image.

The small-sized insect pests in this study can be distinguished more accurately at a small scale as opposed to a global (i.e., whole RoI) image. Thus, the RoI image is divided into multiple sub-blocks using a sliding window and each block size was 64 × 64 pixels, as shown in Figure 4.

##### Saliency Region Detection

In the sub-block image, small-size insects in local window were regarded as “novelty” objects or saliency regions. These insects can be identified and localized using the saliency region detection method. In this study, a spectral residual model (Zhou and Zhang, 2007) is used to locate the small-size insects in each sub-block image. To construct the saliency map, the spectral residual is extracted by analyzing the log-spectrum of the input sub-block image. Given a sub-block image *I*(*x*), the saliency map image *S*(*x*) can be obtained using the following equations:


(1) A⁢(f)=|F⁢[I⁢(x)]|

(2) P⁢(f)=φ⁢(F⁢[I⁢(x)])

(3) L⁢(f)=log⁡(A⁢(f))

(4) $$R\,\left(f\right)=L\,\left(f\right)-h_{n}\,{\left(f\right)}^{*}\,L\,\left(f\right)$$

(5) $$S\,\left(x\right)=g\,{\left(x\right)}^{*}\,F^{-1}\,{\left[\exp\left(R\,\left(f\right)+i\,P\,\left(f\right)\right)\right]}^{2}$$

where *F* and *F*^−1^ denote the Fourier Transform (FT) and Inverse Fourier Transform (IFT), respectively. *A*(*f*) and *P*(*f*) denote the amplitude and phase spectrum of the image, respectively. *L*(*f*) and *R*(*f*) denote the log spectrum and spectral residual. *h*_*n*_(*f*) and *g*(*x*) denote local average and Gaussian filter, respectively.

**FIGURE 5:**
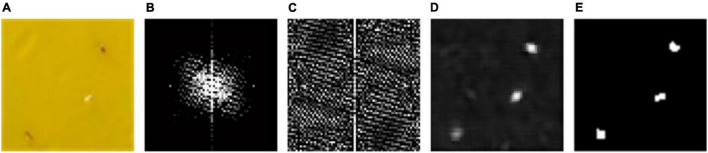
Illustration of saliency region detection for insect pests collected on sticky traps and identified with image acquisition software: **(A)** a sub-block image from the sticky trap, **(B)** log-spectrum distribution of the sub-block, **(C)** the spectral residual image, **(D)** a saliency map of the insect pests and **(E)** binary image of the saliency map.

The pipeline of saliency region detection is illustrated in Figure 5. First, the log-spectrum using two-dimensional fast Fourier transform (2DFFT) and a logarithm to the input sub-block image (Figure 5A) are calculated. As shown in Figure 5B, most of the log-spectrum distribute in the low frequency portion (white regions of the center), which represents the input image includes slowly changing background and a few salient objects. The spectral residual is obtained by the log-spectrum minus the average spectrum which can be approximated using a local average filter (e.g., step size = 3). However, it can be found from Figure 5C that the spectral residual contains high frequency information, which is sharply different from the log-spectrum. After using a two-dimensional inverse Fourier transform (2DIFFT), the saliency map in spatial domain is constructed and the novelty objects (candidate insects in this study) of the image can be seen more clearly in the saliency map (Figure 5D).

##### Image Binarization

**FIGURE 6:**
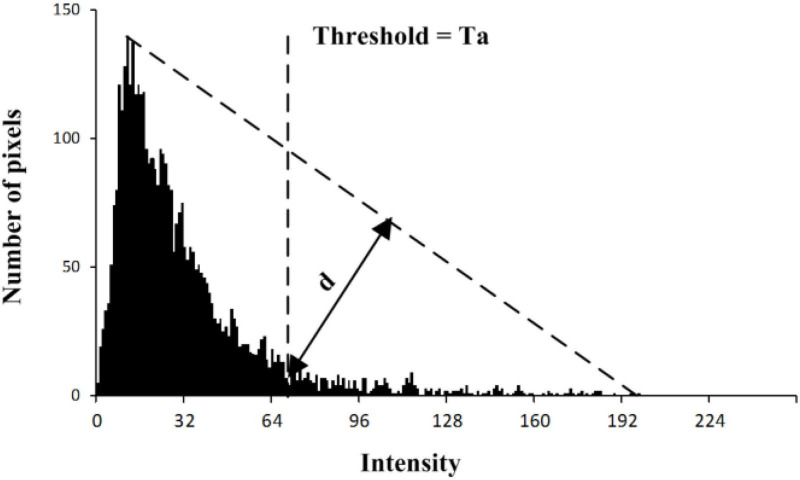
Determination of the threshold value for each saliency map.

The saliency map is an explicit representation of candidate insects in the image. Furthermore, there may be multiple objects within a saliency region. In this section, a threshold segmentation method combined with watershed theory (Meyer, 1994; Dorj et al., 2017) is designed to detect insects within this saliency region. First, the saliency map image is transformed into a binary image using an adaptive threshold value and then watershed algorithm (Tarabalka et al., 2010; Zhang et al., 2014) is selected to segment multiple objects. Since the intensity of the histogram of the saliency map only had a peak and the peak is close to the darkest side, as shown in Figure 6, the threshold value is adaptively determined by using a triangle theory. The steps are as followed:

S1: Constructing a line from the peak to the first darkest point on the intensity histogram.

S2: Calculating the distance from each point of histogram to the line.

S3: The location Ta which has the largest distance *d* is the threshold value.

A binary image could be obtained by using the proposed threshold method. Furthermore, the size of target pests is approximately from 5 pixels to 25 pixels in a sub-block image. Therefore, non-target objects whose sizes are less than 5 pixels or more than 25 pixels are removed from the binary image. Ultimately, the remaining isolated individuals represent the location results (Figure 5E).

#### Feature Extraction

**FIGURE 7:**
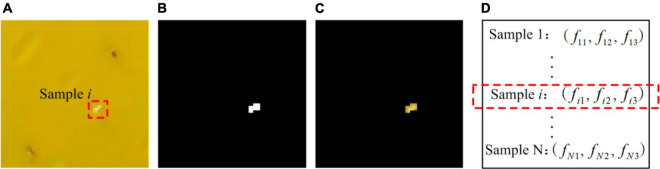
Images documenting feature extraction of individual insects. **(A)** Sub-block image, **(B)** a detected region, **(C)** an isolated insect, **(D)** feature vector in color space.

To identify insect species on the RoI image, all isolated insects are segmented and their features are extracted from sub-block images. As shown in Figure 7, the sample pest *i* on a sub-block image (Figure 7A) could be segmented into an isolated pest (Figure 7C) by performing a logical conjunction operation between the sub-block image and the detected region (Figure 7B). As shown in Figure 7C, the shape of segmented object is different from its original appearance because of inaccurate segmentation for some pixels of the insects, especially in the boundary of insect region. Therefore, the insect contours are not smooth and the insects can’t be accurately identified based solely on shape feature. However, for the two species (whitefly and thrips), different color variation occurs as shown in Figure 4B. Therefore, the color feature is a critical factor to identify the insect species. To determine the optimal color feature, four color models widely used in computer vision-based applications (Kurtulmus et al., 2011; Hu et al., 2012; Reyes et al., 2017; Tan et al., 2018) are evaluated: RGB (red, green and blue), HSV (hue, saturation and value), YCbCr (luminance, blue-difference and red-difference) and L*a*b* (lightness, green-red, and blue-yellow).

The features of each segmented sample are represented by average values of R, G, and B components in RGB space, H, S, and V component in HSV space, Y, Cb, and Cr components in YCbCr space, L*, a*, and b* in L*a*b* color space, respectively. The transformations are shown in Eqs (6)–(9).


(6) $$\bar{R}=\frac{\sum_{i=1}^{n_{j}}R_{\text{i}}}{n_{j}},\bar{G}=\frac{\sum_{i=1}^{n_{j}}G_{\text{i}}}{n_{j}},\bar{B}=\frac{\sum_{i=1}^{n_{j}}B_{\text{i}}}{n_{j}}$$

(7) $$\bar{H}=\frac{\sum_{i=1}^{n_{j}}H_{\text{i}}}{n_{j}},\bar{S}=\frac{\sum_{i=1}^{n_{j}}S_{\text{i}}}{n_{j}},\bar{V}=\frac{\sum_{i=1}^{n_{j}}V_{\text{i}}}{n_{j}}$$

(8) $$\bar{Y}=\frac{\sum_{i=1}^{n_{j}}Y_{\text{i}}}{n_{j}},\bar{C\,b}=\frac{\sum_{i=1}^{n_{j}}C\,b_{\text{i}}}{n_{j}},\bar{C\,r}=\frac{\sum_{i=1}^{n_{j}}C\,r_{\text{i}}}{n_{j}}$$

(9) $$\bar{L^{{}^{*}}}=\frac{\sum_{i=1}^{n_{j}}L_{\text{i}}^{{}^{*}}}{n_{j}},\bar{a^{{}^{*}}}=\frac{\sum_{i=1}^{n_{j}}a_{\text{i}}^{{}^{*}}}{n_{j}},\bar{b^{{}^{*}}}=\frac{\sum_{i=1}^{n_{j}}b_{\text{i}}^{{}^{*}}}{n_{j}}$$

where $\bar{R}$, $\bar{G}$, $\bar{B}$, $\bar{H}$, $\bar{S}$, $\bar{V}$, $\bar{Y}$, $\bar{C\,b}$, $\bar{C\,r}$$\bar{L^{*}}$, $\bar{a^{*}}$, and $\bar{b^{*}}$ denote the average value of corresponding color component over all pixels. *n*_*j*_ denotes the number of image pixel of the *j*th segmented insect sample. The three average components of a sample in each color space constructed a three-dimensional vector f_*i*1_,*f*_*i*2_,*f*_*i*3_, as shown in Figure 7D, which is used as the input of the classifier (discussed in Section “Multi-Class Recognition Model”) for species classification.

#### Multi-Class Recognition Model

After features extraction, a following step is to develop an efficient model to identify different insect species. In this study, the supervised learning model, support vector machine (SVM) (Chen et al., 2010; Li et al., 2010; Saruta et al., 2013), is used as a classifier to discriminate objects between whitefly, thrips or background. For the SVM model, all samples are viewed as points in p-dimensional space and these points in separate categories are divided through a clear gap that is as wide as possible (Rumpf et al., 2010). New examples are then mapped into the same space and predicted to a certain category based on which side of the gap they fall (Larese et al., 2014). In this study, each sample in the training set is marked as belonging to a whitefly, a thrips or background object and all samples are formed into pairs of features-label examples such {x*_i_*,*y*_*i*_}, where x*_i_* is the three-dimensional feature vector and *y*_*i*_ is a class label. Our ultimate goal is to find the “maximum-margin hyperplane” that can divide the groups of samples. One of many possible hyperplanes can be expressed by the following equation:


(10) $$f\,\left({\text{x}}_{i}\right)=w^{T}\,{\text{x}}_{i}+b=0$$

where *w* ∈ *R^d^* and *b* ∈ *R*. A support vector classifier selects the hyperplane that maximizes the margin. This optimization problem can be posed as follows:


(11) $$\min_{w,b}||w||,y_{i}\,\left(w^{T}\,x_{i}+b\right)-1\geq 0$$

In this study, the LIBSVM package (Chang and Lin, 2015), which supports support vector classification (C-SVC, mu-SVC) and regression (epsilon SVR, nu-SVR), is used to conduct the identification model development.

### Performance Evaluation

The detection results are evaluated using metrics, such as the true positive rate (TPR), false positive rate (FPR) and detection accuracy. These metrics have been widely used in object classification and detection areas (Xia et al., 2012; Nasirahmadi et al., 2017; Shrestha et al., 2018). TPR refers to the effectiveness of a classifier to identify positive samples, whitefly and thrips in this study. A high TPR value means that most of the positive samples are detected successfully. While FPR indicates that how effectively a classifier could identify negative samples. A low FPR value indicates the identification results contain a low percentage of false alarms and a high percentage of true positives. These parameters are calculated as follows:


(12) $$T\,P\,R=\frac{T\,P}{T\,P+F\,N}$$

(13) $$F\,P\,R=\frac{F\,P}{T\,N+F\,P}$$

(14) $$A\,\text{ccu}\,r\,a\,c\,y=\frac{T\,P+T\,N}{T\,P+T\,N+F\,P+F\,N}$$

where TP, TN, FP, and FN denote true positive (correctly identified), true negative (correctly rejected), false positive (incorrectly identified) and false negative (incorrectly rejected), respectively.

## Results

### Sample Distribution in Different Color Space

**FIGURE 8:**
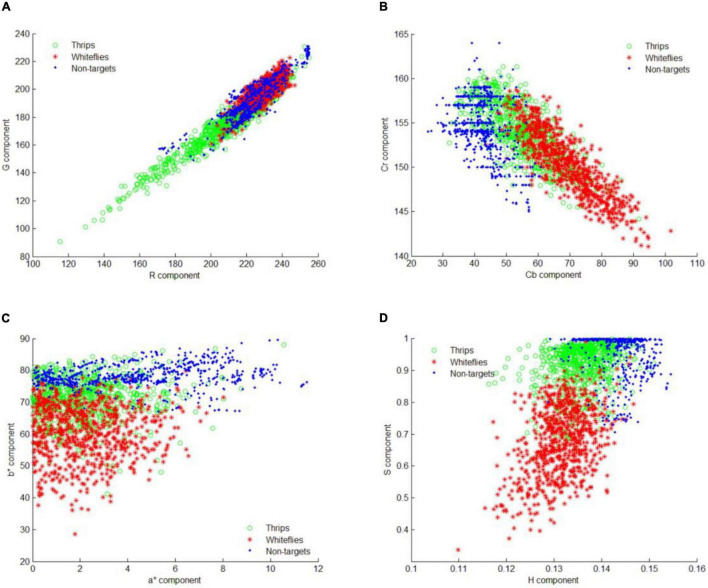
Feature distribution of all the training samples. **(A)** Sample distribution in RGB color space, **(B)** sample distribution in L*a*b* color space, **(C)** sample distribution in YCbCr color space, and **(D)** sample distribution in HSV color space.

After saliency region detector scanning across all images, the locations of most potential objects are detected. To identify those objects into different species, the feature distribution of whitefly, thrips and background are analyzed in four color spaces. The component of R, G, a*, b*, Cb, Cr, H, S in RGB, L*a*b*, YCbCr and HSV color space are illustrated in Figure 8. The distributions of different features showed that there is considerable overlap between targets (whitefly and thrips) and background in the RGB color feature space. Therefore, it is difficult to classify whitefly and thrips from the background category (Figure 8A). As shown in Figure 8B, whitefly can be separated from background category in L*a*b* color space but thrips still can’t be separated from background category. Furthermore, the distribution of YCbCr features was similar to L*a*b* color space and thrips can’t be separated from category. In addition, there is some confusion between whitefly and thrips (Figure 8C). Figure 8D documents the distribution of the three categories in HSV color space, which shows that it is relatively easy to classify the three categories. Therefore, the components of H, S and V are used to detect different insect species in current study.

### Detection Results

**FIGURE 9:**
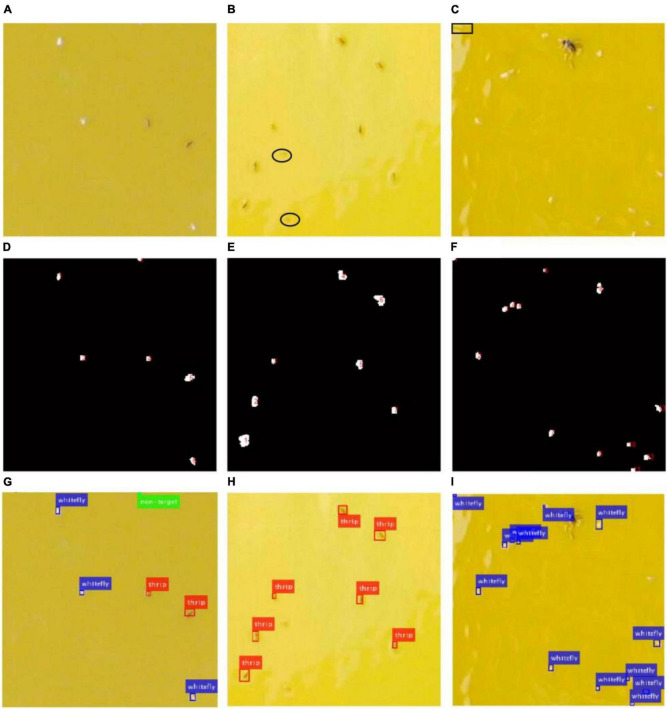
Original images, location results and detection results of three image samples with different quality. **(A,D,G)** Are for sample 1, **(B,E,H)** are for sample 2, **(C,F,I)** are for sample 3.

The images captured from the field are complicated due to variable conditions such as unstable illumination, light reflection and various objects. Figure 9 shows some examples of insect detection of different species in three sub-blocked images with different image quality.

As shown in Figure 9A, it is a good-quality image with smooth background. However, most of background in Figure 9B is whitened because of the sticky glue degenerated over time, and light reflection causes low-quality image in Figure 9C, which brings difficulties to the insect detection. The location results using the saliency region detection method are numbered as shown in Figures 9D–F, respectively. Every identified object is located using a bounding box, red for thrips, blue for whitefly and green for background category (non-target) in Figures 9G–I, respectively. The results showed that all whiteflies and thrips in Figure 9A are detected successfully. However, there were some missing detections marked with black ellipse in Figure 9B. Furthermore, some spots (marked with black rectangle) caused by sticky glue are falsely classified as whiteflies in Figure 9C.

The insect detection performance is evaluated using TPR, FPR and accuracy which are described in section “Performance Evaluation.” Initially, the two pest species in the testing dataset are separately marked manually and subsequently the evaluation metrics are calculated according to the detection results using Eqs (11)–(13). The overall detection performance on the three categories is shown in Table 1. The TPRs for whitefly and background categories were over 90% and the lowest TPR rate of 80.1% is obtained by the thrips category. The reason may be that some insects are attached to the sticky traps for a long time, and they became obscure due to weathering and dryness causing lack of detection. Additionally, the size of thrips is particularly small, ranging from 5 pixels to 20 pixels, such that it merged with the background thereby becoming indistinct. The feature distribution between the background and thrips in section “Sample Distribution in Different Color Space” may further verify the result. However, these recently trapped insects are easier to locate and identify.

**TABLE 1:** Detection performance for small-size pests (whitefly and thrips) by the SVM classifier using field sticky trap images (*n* = 18, mean ± *SD*).

Objects	Performance metrics		
	
	TPR	FPR	Accuracy
Whitefly Thrips Background	0.931 ± 0.031 0.801 ± 0.037 0.930 ± 0.021	0.099 ± 0.019 0.123 ± 0.039 0.116 ± 0.037	0.939 ± 0.015 0.898 ± 0.022 0.933 ± 0.014

The detection method for all categories produced false positives. The lowest FPR of 9.9% is for whitefly but is higher for thrips (12.3%) and background detection (11.6%). These are typically caused by degeneration of glue on the sticky trap and these produced “noise” in the form of point, stripe and bulk spot. The latter two noises could be easily filtered by this proposed location method. However, spot noises are easier misclassified into pest targets, especially whiteflies due to their size and color being similar to the targets.

**FIGURE 10:**
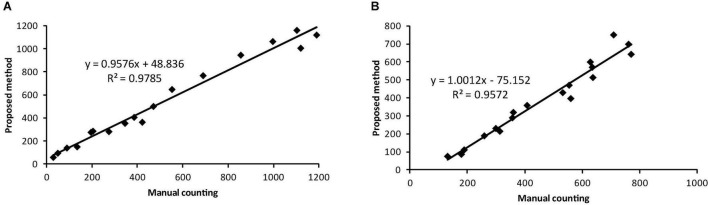
Comparison of results between the proposed detection method and manual counting for **(A)** whitefly and **(B)** thrips using the testing dataset.

The accuracy metric for whitefly is the highest at 93.9% followed by 93.3% for background category and 89.8% for thrips. The identification accuracy is further evaluated by correlation analysis between the proposed method and manual counting, as shown in Figure 10. The coefficient of determination, *R*^2^, reached values of 0.9785 and 0.9572 for whitefly and thrips in the test dataset, respectively. Compared with manual counting, the proposed detection algorithm tended to overestimate the abundance of whitefly and underestimate thrips. Additionally, there are higher FPR for whitefly and increased TPR for thrips in the test dataset.

## Discussion

### Principle and Feasibility Analysis

This study clearly demonstrates the utility of using a remote imaging approach combining image processing and pattern recognition technology to locate and identify whitefly and thrips on sticky trap in greenhouse conditions. The detection of whitefly and thrips on the sticky trap is primarily composed of two procedures: candidate target location and subsequent identification. Compared with detection in a large image, the small-sized whiteflies and thrips are more accurately recorded on small visual areas. The image blocking procedure is included in the study to split original image into small sub-blocking images to increase area occupancy rate. From the perspective of information theory, an image consists of two parts: the novelty part (saliency regions) and redundant information (Zhou and Zhang, 2007). The background in a sub-blocking image is the statistical redundant component and whitefly and thrips in the image could be regarded as the novelty component. There are different spectral responses for the novelty and redundant parts of the frequency domain. After removing the frequency response of the redundant part from the whole spectrum, the novelty part can be obtained. The most important advantage is that the saliency region detection model is independent of species, features, or other forms of prior knowledge of the objects.

The second step after object location is multi-class identification. The segmented objects in the first step not only contain whitefly and thrips, but also include the non-target category. However, the identification of whiteflies and thrips from non-targets is challenging and feature extraction is a key step in the classification process. Similar studies on the insect detection extracted shape features such as size, body eccentricity and solidity to classify species (Solis-Sánchez et al., 2011; Wang et al., 2012; Espinoza et al., 2016). However, due to the small size characteristics of whiteflies and thrips, the contours of the pests are not smooth after they are extracted from background and could not be accurately identified based on shape features. Despite the challenges, color feature analysis revealed different feature distribution in HSV color space and three color components (H, S, and V) are used as feature input of SVM classifier to identify whiteflies and thrips in this study.

### Robustness Analysis

**FIGURE 11:**
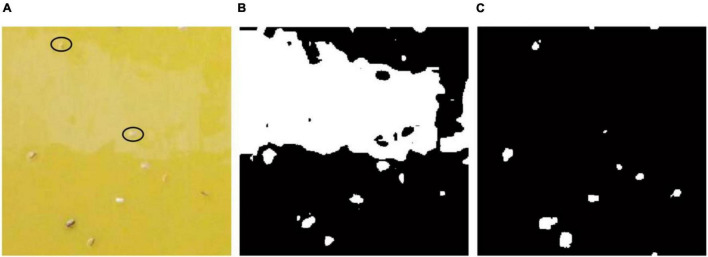
Pest segmentation results of a sub-blocking image with noise by different methods. **(A)** Original image, **(B)** segmentation result of YCbCr color model, **(C)** segmentation result of the proposed method.

The image-based pest identification method has previously demonstrated high performance on collected images in the laboratory conditions (Cho et al., 2007; Boissard et al., 2008). However, field condition are very different from the laboratory environment since the sticky trap images captured in greenhouse can be influenced by various factors including sticky glue degeneration, light reflection and unstable variable illumination conditions (Xia et al., 2012). For example, Cho et al. (Cho et al., 2007) utilized the RGB and YUV color model to separate three different species. In addition, insect segmentation by YCbCr color model has revealed better results than other methods among different color models (Xia et al., 2015), but these segmentation methods based on the color model have some shortcomings when applied into field images. As shown in Figure 11A, there is some noise in upper part of the image caused by degeneration of sticky glue and light reflection. The segmentation result (Figure 11B) using the YCbCr color model shows these objects (marked with black ellipse in Figure 11A) are entirely missed. However, these objects in the noise region still can be segmented by the proposed method (Figure 11C). Although the multifractal analysis method was designed against noise when used under field conditions and showed high performance regarding accuracy, only one species of pest, whiteflies, had been detected and the image collected device and procedure was relatively complex (Xia et al., 2012). Rather than directly counting the pests captured on the traps, Sun et al. (2017) treated trapped pests as noise with 2DFT serving as a noise collector. This method obtained a high correlation with human counting when there was no other noise, but the Fourier transform in a case when there are noise and pests at low population density is similar to another case when pests at high population density and no noise. In addition, it could not address the problem associated with multi-class identification.

In current study, the pests are regarded as novelty objects and located by the saliency region detection method which is independent of color features and other forms of prior knowledge of the objects. Therefore, good robustness of pest segmentation in field images could be obtained by the proposed method.

Conversely, since some pests are attached to the trap for a long time, there is limited resolution in the imaging and the pest region in the saliency map is unclear, which will cause missing detection after binary image processing. Contrasting with the Otsu algorithm (Otsu, 1979), the threshold selected by the triangle method (section “Candidate Object Location”) can improve the detection rate since it utilized the single-peaked feature of a histogram, but there are still some pests with low novelty that are not reliably detected. In actual application, the optimal option is replacement of the sticky trap on schedule to avoid loss of resolution and missing data due to sticky trap degeneration.

### Comparisons With Previous Methods

Regarding to insect pest detection using sticky traps, several image-based methods had been reported, including handcrafted feature-based and deep learning-based methods. However, it is difficult to compare the performances of these previous studies with the proposed one quantitatively because of the use of different dataset which is not publicly available. Therefore, a qualitative analysis had been made in this study. Comparisons of the proposed approach with some methods for detecting greenhouse pests, such as whitefly and thrips, using sticky trap images are summarized in Table 2. Two previous method proposed by Xia et al (2015) and Espinoza et al (2016) used images scanned in the laboratory as research materials, but the comparison showed that the prediction results of the proposed method outperformed the method of Xia et al (2015). While the detection results reported by Espinoza et al (2016) presented the higher accuracy, the study used thresholding method to segment targets, which causes the results were likely influenced by the segmentation threshold. Qiao et al (2008) reported a fact that a small threshold loses relevant information, while a large threshold produces more noise, so its accuracy is much lower than that of the proposed method. It must be acknowledged that the performance of the proposed model is lower than that of deep-learning-based method reported by Li et al (2021), however, the method based on deep learning technology has high complexity and depends on high-performance hardware, such as GPUs^1^.

**TABLE 2:** Comparison between the proposed and previous methods for detection of whitefly and thrips using sticky trap images.

Method	Imaging scene	Segmentation	Features	Classification method	Pest species	Average accuracy (%)
Qiao et al. (2008)	Field-based	Thresholding	Color and size	Comparative method	Whitefly	76.9
Xia et al. (2015)	Lab-based	Thresholding	Color and size	Mahalanobis distance	Whitefly, aphids, thrips	91.0
Espinoza et al. (2016)	Lab-based	Thresholding	Morphology and color	ANN	Whitefly and thrips	94.0
Li et al. (2021)	Field-based	No	Deep learning automatically	Softmax	Whitefly and thrips	94.4
The proposed method	Field-based	Spectral residual model	Color	SVM	Whitefly and thrips	91.9

### Pest Identification and Management

During our experiments in a greenhouse planted with pepper, whitely and thrips are the two main pests. Although only whitefly and thrips are identified in this study, the proposed method can have additional applications into the detection of multiple pests in greenhouse agriculture. The methodology for the detection of more than three species is similar to that proposed in section “Detection Method” except that more categories will be required to extract information to allow for the construction of a new baseline dataset.

In ecological studies, IPM usually relies on pest population density assessment in a given area and is often estimated based on trap counts (Petrovskii et al., 2012; Pinto-Zevallos and Vänninen, 2013). Therefore, precision identification and counting of pests in a sticky trap image is of critical importance for the estimation of population density. However, the relationship between trap counts of whitefly and thrips and the actual population density in the greenhouse is not clear. Such validation studies would form a critical future basis for pest management using image processing of pest populations in greenhouses (or open field situations).

## Conclusion

This study proposed a novel approach for the detection of adult-stage whiteflies and thrips on sticky traps in greenhouses. The approach consisted of three modules: object location, feature extraction and multi-class recognition. The sticky trap image was divided into sub-block images and novelty objects within each sub-block image were located using a saliency region detection model. Furthermore, average values of three components in HSV color space were extracted to train a SVM classifier. Ultimately, HSV color features were calculated and used as input of the trained SVM model to identify whether a detected object was a whitefly or a thrips.

The study shows that adult thrips can be identified with a TPR of 80.1%, FPR of 12.3% and accuracy of 89.8%. Better performance is attained for the identification of whitefly, with a value of 93.1% for TPR, 9.9% for FPR, and 93.9% for accuracy. The proposed method in this study provides the possibility of counting different species of pests in greenhouse conditions by an automated pipeline, alleviating the time-consuming and inaccurate approach associated with grower-based identification of minute insect pests. The findings of the study contribute valuable information pertaining to population density estimation of small insect pests in greenhouse conditions and have broad utility to other systems allowing for decision making processes regarding integrated pest management approaches.

## Data Availability Statement

The raw data supporting the conclusions of this article can be obtained after consulting the corresponding author, without undue reservation.

## Author Contributions

WL and TZ conceived and explored literature. ZY and JL analyzed data. WL wrote the manuscript. ML and CS reviewed and edited the manuscript. All authors read and approved the manuscript.

## Conflict of Interest

The authors declare that the research was conducted in the absence of any commercial or financial relationships that could be construed as a potential conflict of interest.

## Publisher’s Note

All claims expressed in this article are solely those of the authors and do not necessarily represent those of their affiliated organizations, or those of the publisher, the editors and the reviewers. Any product that may be evaluated in this article, or claim that may be made by its manufacturer, is not guaranteed or endorsed by the publisher.

## Footnotes

1
https://www.nvidia.cn/
